# Comparison of preemptive effect of intravenous ketorolac versus meperidine on postoperative shivering and pain  in patients undergoing cesarean section under spinal anesthesia: A prospective, randomized, double-blind study

**DOI:** 10.22088/cjim.9.2.151

**Published:** 2018

**Authors:** Marzieh Beigom Khezri, Maryam Al-sadat Mosallaei, Mehdi Ebtehaj, Navid Mohammadi

**Affiliations:** 1 Department of Anesthesiology, Faculty of Medicine, Qazvin University of Medical Sciences, Qazvin, Iran.; 2 Preventive Medicine and Public Health Research Center, Iran University of Medical Sciences, Tehran, Iran.

**Keywords:** Shivering, Ketorolac, Meperidine, Spinal anesthesia, Cesarean section

## Abstract

**Background:**

Pain and shivering are two unpleasant problems in postoperative period. Various techniques are used to alleviate the postoperative shivering and pain. We compared the preemptive prescription of a single dose of intravenous meperidine and ketorolac on postoperative pain and shivering in patients undergoing cesarean section with spinal anesthesia.

**Methods:**

One hundred and fifty patients who were scheduled for elective cesarean section under spinal anesthesia were randomly allocated to one of three study groups to receive intravenous ketorolac (group K), meperidine (group M) or normal saline (group P). Time to first analgesic request, analgesic requirement in the first 24 hours after surgery, body tympanic temperature, hemodynamic variables and incidence of shivering were assessed as outcome variables.

**Results:**

There was no significant difference between meperidine and ketorolac groups in terms of prevalence of shivering, although both groups were different from the placebo group (p<0.04). The mean time to first analgesic request was longer in group k (3.8±1.4) and groups M (3.3±1.2) than in group P (2.1±0.8) hours (p<0.001).

**Conclusions:**

The preemptive prescription of a single dose of intravenous meperidine and ketorolac can provide a satisfying analgesia immediately after surgery and decrease shivering prevalence without any serious side effects.

Postoperative pain and postanesthetic shivering remain two common problems after surgery. These problems should be managed to improve the outcome and satisfaction of patients. In a review of 21 studies, median incidence of shivering related to regional anesthesia was reported as 55% (1, 2). Shivering may increase cardiac output, circulating catecholamine’s, intracranial and intraocular pressures, and blood pressure (3,4). Furthermore, it is considered as a responsible factor for exacerbating postoperative pain and patient discomfort (5, 6). Pain control after cesarean section improves breastfeeding and mother satisfaction. In addition, postoperative pain is associated with neuroendocrine responses (6). It is believed that central sensitization is one of the mechanisms implicated in chronic postoperative pain (7, 8). Postoperative pain could be a provocative factor for postoperative shivering and its appropriate treatment prevents non-thermoregulatory tremors (9-11). Shivering also causes aggravating of postoperative pain by stretching of sutures. Painful stimulation slightly increases the vasoconstriction threshold anesthesia (11). Several techniques are used for the prevention and treatment of postoperative pain and shivering, such as administration of meperidine, buspirone, nefopam, clonidine, alfentanil, dolasetron, ketanserin, doxapram, and dexmedetomidine (12-15).

Meperidine is widely used to treat postoperative shivering and pain (4, 12, 13). Although its mechanism of action is not completely clear, meperidine probably acts directly on the thermoregulatory center or via agonistic effect on μ and κ-opioid receptors (4, 16, 17). Like many other narcotics, meperidine has various side-effects such as respiratory depression, hypotension, tachycardia, nausea, vomiting, itching, decreased gastrointestinal (GI) motility, and physical dependency (17). Furthermore, it is reported that only a single administration of an opioid may also induce a long-lasting reduction of threshold of pain sensitivity, which leads to delayed hyperalgesia and somewhat increased incidence of postoperative shivering (18-21). Therefore, the potential clinical advantages of new drugs in this setting need to be evaluated.

It is proposed that steroidal and non-steroidal anti-inflammatory agents (NSAIDS) prevent postoperative shivering through either reduction of postoperative pain or inhibition of releasing vasoconstrictor and pyrogenic cytokines (6, 22-26). Ketorolac (NSAIDs category) has analgesic properties through direct anti-inflammatory effects. Moreover, ketorolac neither causes respiratory depression, nor other side-effects such as vomiting, itching, hyperalgesia effect, and hemodynamic instability, although, it has some gastrointestinal and antiplatelet effects (27). We hypothesized that ketorolac may relieve pain and shivering after cesarean section without pruritus, respiratory depression, hemodynamic instability, or hyperalgesia, (at least as much as meperidine). In order to test our hypothesis, we designed a randomized, double-blind, placebo-controlled study to compare the postoperative analgesic and anti-shivering effects of ketorolac and pethidine in patients undergoing cesarean section under spinal anesthesia.

## Methods

 This trial has been registered in Iranian Clinical Trial Registry (IRCT201104073051N4). Following approval of ethics committee and informed patient’s consent, a randomized study was started with one hundred and fifty adult patients. All patients had physical status I and II according to American Society of Anesthesiologists (ASA) aged 18 to 40 years. They were also scheduled from October 2014 to November 2015 for elective cesarean section under spinal anesthesia at Kowsar Hospital which is a referral Obstetrics/Gynecology center in Qazvin, Iran. Patients with ASA physical status III or IV, cardiac arrhythmias, myocardial insufficiency, body temperature >37.5°C, muscle diseases, Parkinson disease, administration of opioids for long term treatment, history of hypersensitivity to nonsteroidal and steroidal anti-inflammatory agents and peptic ulcer were excluded. Randomization was undertaken by means of computer generated random number in sealed opaque envelopes. Blinding was achieved using equal amounts of drugs (2 mL), while each syringe was labeled as A, B, and C per its content. Identical coded syringes prepared by the personnel (who were not involved in the study) were randomly handed to the anesthetists, who were unaware of the identity of the drug formulations. The patients were randomly divided into three groups of 50 each. Ten minutes before the spinal anesthesia, group K received 30 mg ketorolac, group M received 50 mg meperidine and group P received 2 ml normal saline intravenously.

 Thermometry from tympanic membrane was done 10 min before anesthesia and every 30 minutes after surgery up to an hour. Temperature of operating room was approximately 22°C. All patients received 5-7 ml/kg lactated Ringer’s solution before spinal anesthesia. After using an aseptic technique, a 25-gauge Quincke needle was inserted intrathecally via a midline approach into the L4-5 interspaces, while the patient was in a sitting position with 2.5 ml bupivacaine 0.5% in all three groups. The primary outcomes were to evaluate the time to first requirement of analgesic supplement, total analgesic consumption in the first 24 hours after surgery, and incidence and severity of postoperative shivering. Postoperative analgesia was defined as the time from intrathecal injection of the anesthetic solution to the first request for analgesics. Patients were trained preoperatively for the use of the visual analogue scale (VAS) of pain from zero to 10 (0 no pain, 10 maximum imaginable pain) for pain assessment. 

If the VAS exceeded four and the patient needed a supplement analgesic, apotel 500 mg was given intravenously as postoperative pain relief. Shivering was classified as mild (muscular activity in only one muscle group), moderate (muscular activity in more than one muscle group but not generalized) and severe (shivering involving whole body). After the end of any surgery, shivering scores were assessed by an anesthesiologist resident who was unaware of the patients’ assignment in the PACU (Post Anesthesia Care Unit) for a minimum of 60 minutes for observation. If shivering was severe, 8 mg of ondansetrone was given intravenously (IV). The secondary outcomes included hemodynamic variables.

To calculate the sample size, data from previous similar studies were taken into consideration (2, 6). A sample size of 40 patients per group were required to provide 90% power to detect a reduction of the incidence of post-anesthetic shivering from 50% to 25%. To compensate for dropout cases and shifting from normality in data distribution, 50 cases were recruited in each group. Normal distribution of the data was tested by Kolmogorov-Smirnov test. Normally, distributed data were expressed as means and standard deviations (SD). Analysis of variance (ANOVA) and repeated measure analysis were used for continuous parametric variables. Within groups, comparisons were made using the Tukey's post-hoc analysis.  Chi-square test was used for comparing the incidence of shivering between the groups. A p<0.05 was considered as statistically significant. Statistical analysis was carried out using SPSS Version 16 for Windows (SPSS, Chicago, IL).

## Results

One hundred and sixty patients were screened and 150 who met the eligibility criteria were enrolled, consented and randomized.

**Figure 1:**
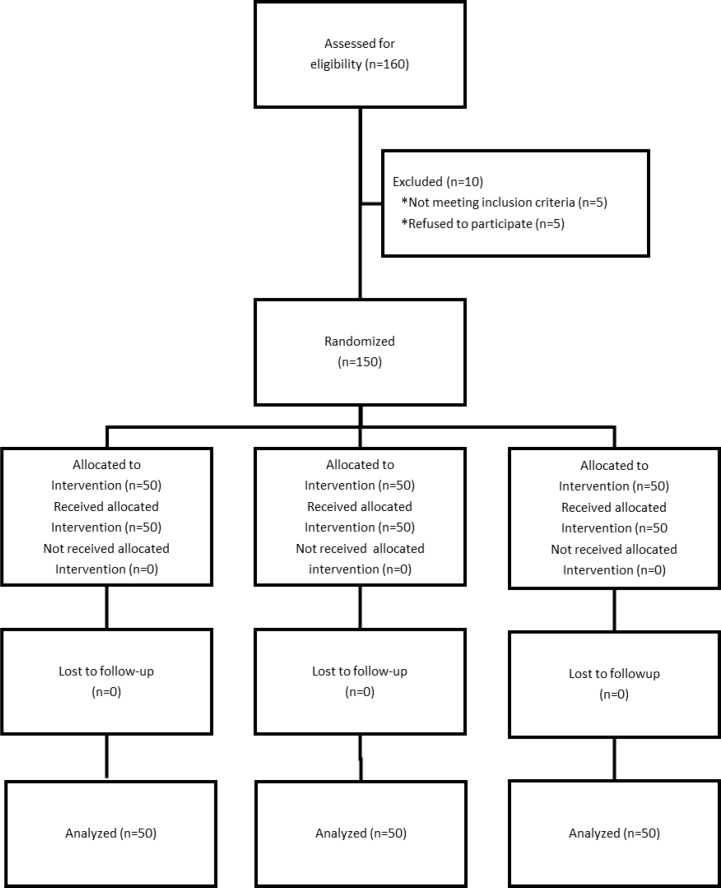
Consort flow diagram

All enrolled patients completed the trial (figure 1). 

Three groups were similar in terms of age, weight, height, ASA physical status and duration of surgery (table 1). None of the patients received transfusion. As shown in table 2, there was a significant difference between three groups in the overall incidence of shivering for patients sixty minutes after surgery (P=0.041). The incidence of shivering after sixty minutes of surgery was 60% in group K, 66% in group M and 82% in group P. Meanwhile there was a significant difference between the three groups in the severity of shivering during the first sixty minutes after the end of surgery (P=0.012). The mean temperature changes in the groups K, M and P were (0.31±0.11C), (0.67±0.09C) and (0.71±0.01C), respectively. This difference was found to be significant among all three groups (P<0.001).

**Table 1:** Demographic data associated with the study

**Groups**	**Ketorolac** **(N=50)** **Mean±SD**	**Meperidine** **(N=50)** **Mean±SD**	**Placebo** **(N=50)** **Mean±SD**	**P-** **value**
Weight(kg)	75.21±5.96	72.45±6.59	74.75±6.88	0.169
Height(cm)	160.98±2.9	1.9±160.1	2.1±160.56	0.409
Age(years)	6.9±27.4	6.1±27.9	7.8±28.3	0.801
Duration of surgery(min)	85.63±15.70	79.16±20.11	81.70±18.76	0.840

**Table 2:** Incidence and severity of shivering

**Shivering**	**Ketorolac** **N=50**	**Meperidine** **N=50**	**Placebo** **N=50**	**P value**
Incidence, n (%)	30(%60)	33(%66)	41(%82)	0.041
Severity (a, b, c,)	4,8,18	8,12,13	2,10,29	0.012
Treatment (Ondansetron)requirement	18(%60)	13(%39.4)	29(%70.7)	0.042

Values are number (percent age) of patients. a) mild shivering, b) moderate shivering, c) severe shivering.

**Figure 2:**
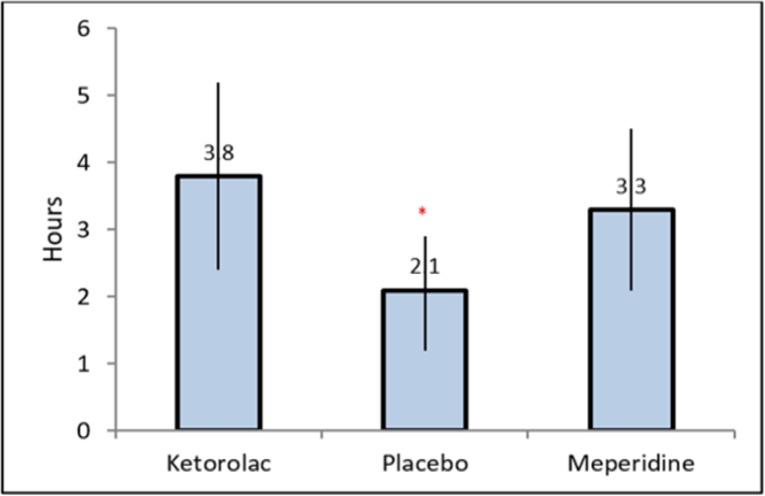
Comparison of analgesic duration in three study groups

As shown in figure 2, the mean time to first analgesic request was significantly longer in groups K (3.8±1.4) and M (3.3±1.2) compared to group P (2.1±0.8 hours) (p<0.001 for both comparisons). However, the difference between groups K and M was insignificant (*P*=0.06) through Tukey's post hoc test. The total analgesic consumption by patients during the first 24 hours after surgery in ketorolac and meperidine groups was significantly smaller than in control group (*P<*0.001) while we failed to find a significant difference between K and M groups (*P*=0.41). There was no respiratory depression, excessive blood loss, and urinary retention in our patients and the SPO2 was in the normal range. 

**Figure 3:**
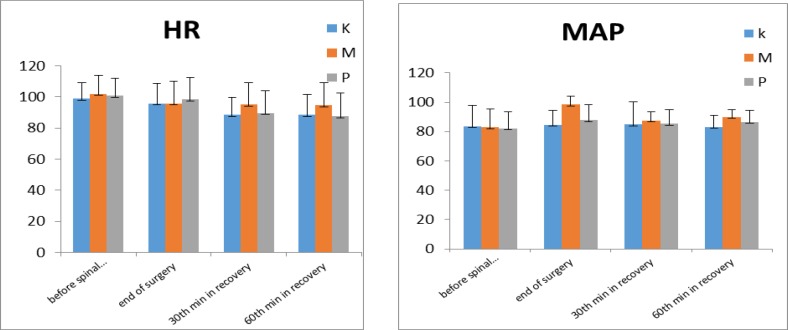
Hemodynamic changes including heart rate (bpm) (HR) and mean arterial blood pressure (mmHg) (MAP)

Figure 3 shows that the mean arterial pressure (MAP) variation between three groups was significant (P=0.001), while the difference between ketorolac and meperidine groups *was* insignificant *(P*=*0.42)*. *The m*ean difference of heart rate (HR) variation was also significant between three groups (P=0.001). Neither gastrointestinal problems, respiratory complication nor postoperative hemorrhage (due to platelet aggregation disturbances) were reported in patients.

Data were expressed as mean and error bars representing SD deviation values. *Significant difference between the three groups.

K=Ketorolac, M= Meperidine, P=Placebo. Data are expressed  as mean and error bars representing standard deviation.

## Discussion

**).**

**finding which should be taken into account is** **However, our study had some limitations; we did not evaluate the dose–response or the effect of multiple dose therapy.** **Good pain** ***control*** **after surgery is important to** ***prevent* persistent postsurgical pain (**7**, **30**). Further studies are required to evaluate the effect of multiple dose administration of ketorolac to find methods to prevent** **the shivering and postsurgical** ***pain*.**

## References

[1] Crowley LJ, Buggy DJ (2008). Shivering and neuraxial anesthesia. Reg Anesth Pain Med.

[2] Roy JD, Girard M, Drolet P (2004). Intrathecal meperidine decreases shivering during cesarean delivery under spinal anesthesia. Anesth Analg.

[3] Usta B, Gozdemir M, Demircioglu RI (2011). Dexmedetomidine for the prevention of shivering during spinal anesthesia. Clinics (Sao Paulo).

[4] Eydi M, Golzari SE, Aghamohammadi D (2014). Postoperative management of shivering: a comparison of pethidine vs. ketamine. Anesth Pain Med.

[5] Kose EA, Dal D, Akinci SB, Saricaoglu F, Aypar U (2008). The efficacy of ketamine for the treatment of postoperative shivering. Anesth Analg.

[6] Khezri MB, Bandari AM, Asefzade S, Atlasbaf A (2011). The effect of diclofenac Na supp on postoperative shivering in patients undergoing elective cesarean section surgery. Pak J Med Sci.

[7] Bennett EE, Walsh KM, Thompson NR, Krishnaney AA (2017). Central Sensitization Inventory as a predictor of worse quality of life measures and increased length of stay following spinal fusion. World Neurosurg.

[8] Yaghoobi S, Seddighi M, Yazdi Z, Ghafouri R, Khezri MB (2013). Comparison of postoperative analgesic effect of dexamethasone and fentanyl added to lidocaine through axillary block in forearm fracture. Pain Res Treat.

[9] Horn EP, Schroeder F, Wilhelm S (1999). Postoperative pain facilitates nonthermoregulatory tremor. Anesthesiology.

[10] Panzer O, Ghazanfari N, Sessler DI (1999). Shivering and shivering-like tremor during labor with and without epidural analgesia. Anesthesiology.

[11] Washington DE, Sessler DI, McGuire J (1992). Painful stimulation minimally increases the thermoregulatory threshold for vasoconstriction during enflurane anesthesia in humans. Anesthesiology.

[12] Mokhtarani M, Mahgoub AN, Morioka N (2001). Buspirone and meperidine synergistically reduce the shivering threshold. Anesth Analg.

[13] Doufas AG, Lin CM, Suleman MI (2003). Dexmedetomidine and meperidine additively reduce the shivering threshold in humans. Stroke.

[14] Sessler DI (2003). Treatment: meperidine, clonidine, doxapram, ketanserin, or alfentanil Abolishes short-term postoperative shivering. Can J Anaesth.

[15] Taniguchi Y, Ali SZ, Kimberger O (2010). The effects of nefopam on the gain and maximum intensity of shivering in healthy volunteers. Anesth Analg.

[16] Koczmara C, Perri D, Hyland S, Rousseau L (2005). Meperidine (Demerol®) safety issues. Off J Can Assoc Crit Care Nurs.

[17] Brunton L (2010). Goodman & Gilman's pharmacological basis of therapeutics. 12th ed. McGraw Hill.

[18] Keskin HL, Keskin EA, Avsar AF, Tabuk M, Caglar GS (2003). Pethidine versus tramadol for pain relief during labor. Int J Gynaecol Obstet.

[19] Song YK, Lee C, Seo DH (2014). Interaction between postoperative shivering and hyperalgesia caused by high-dose remifentanil. Korean J Anesthesiol.

[20] Laulin JP, Célèrier E, Larcher A, Le Moal M, Simonnet G (1999). Opiate tolerance to daily heroin administration: apparent phenomenon associated with enhanced pain sensitivity. Neuroscience.

[21] Khezri MB, Rezaei M, Delkhosh Reihany M, Haji Seid Javadi E (2014). Comparison of postoperative analgesic effect of intrathecal clonidine and fentanyl added to bupivacaine in patients undergoing cesarean section: a prospective randomized double-blind study. Pain Res Treat.

[22] Ali Janpour E, Rabiee M, Bani-Hashem N, Jabbari A (2014). Early post-operative relief of pain and shivering using diclofenac suppository versus intravenous pethidine in spinal anesthesia. J Anaesthesiol Clin Pharmacol.

[23] Rostaminezhad A, Karimi Z, Khosravi A, Chohedri A, Ghaffarian Shirazi H (2004). The effect of suppository diclofenac na on post operative shivering in elective caesarian section surgery. Armaghane Danesh Journal.

[24] Pawar MS, Suri N, Kaul N, Lad S, Khan RM (2011). Hydrocortisone reduces postoperative shivering following day care knee arthroscopy. Can J Anaesth.

[25] Abd Elmawgood A, Rashwan S, Rashwan D (2012). Efficacy of prophylactic use of hydrocortisone and low dose ketamine for prevention of shivering during spinal anesthesia. Egyp J Anaesthesia.

[26] Entezarias M, Isazadehfar K (2013). Dexamethasone for prevention of postoperative shivering: a randomized double-blind comparison with pethidine. Int J Prev Med.

[27] Sostres C, Gargallo CJ, Arroyo MT, Lanas A (2010). Adverse effects of non-steroidal anti-inflammatory drugs (NSAIDs, aspirin and coxibs) on upper gastrointestinal tract. Best Pract Res Clin Gastroenterol.

[28] Kundra TS, Kuthiala G, Shrivastava A, Kaur P (2017). A comparative study on the efficacy of dexmedetomidine and tramadol on post‑spinal anesthesia shivering. Saudi J Anaesth.

[29] Shukla U, Malhotra K, Prabhakar T (2011). A comparative study of the effect of clonidine and tramadol on post-spinal anaesthesia shivering. Indian J Anaesth.

[30] Smith LA, Carroll D, Edwards JE, Moore RA, McQuay HJ (2000). Single-dose ketorolac and pethidine in acute postoperative pain: systematic review with meta-analysis. Br J Anaesth.

